# Practical Approaches for the Yeast *Saccharomyces cerevisiae* Genome Modification

**DOI:** 10.3390/ijms241511960

**Published:** 2023-07-26

**Authors:** Elena I. Stepchenkova, Sergey P. Zadorsky, Andrey R. Shumega, Anna Y. Aksenova

**Affiliations:** 1 Department of Genetics and Biotechnology, St. Petersburg State University, 199034 St. Petersburg, Russia; stepchenkova@gmail.com (E.I.S.); zadorsky@mail.ru (S.P.Z.); shumega84@mail.ru (A.R.S.); 2 Vavilov Institute of General Genetics, St. Petersburg Branch, Russian Academy of Sciences, 199034 St. Petersburg, Russia; 3 Laboratory of Amyloid Biology, St. Petersburg State University, 199034 St. Petersburg, Russia

**Keywords:** yeast, *Saccharomyces cerevisiae*, genome modifications, genome editing, yeast transformation, CRISPR/Cas9

## Abstract

The yeast *S. cerevisiae* is a unique genetic object for which a wide range of relatively simple, inexpensive, and non-time-consuming methods have been developed that allow the performing of a wide variety of genome modifications. Among the latter, one can mention point mutations, disruptions and deletions of particular genes and regions of chromosomes, insertion of cassettes for the expression of heterologous genes, targeted chromosomal rearrangements such as translocations and inversions, directed changes in the karyotype (loss or duplication of particular chromosomes, changes in the level of ploidy), mating-type changes, etc. Classical yeast genome manipulations have been advanced with CRISPR/Cas9 technology in recent years that allow for the generation of multiple simultaneous changes in the yeast genome. In this review we discuss practical applications of both the classical yeast genome modification methods as well as CRISPR/Cas9 technology. In addition, we review methods for ploidy changes, including aneuploid generation, methods for mating type switching and directed DSB. Combined with a description of useful selective markers and transformation techniques, this work represents a nearly complete guide to yeast genome modification.

## 1. Introduction

Baker’s yeast *Saccharomyces cerevisiae* is a versatile tool for molecular biology and genetic studies. It is one of the most-studied eukaryotic unicellular organisms with a fully-sequenced and well-annotated genome [1,2]. The yeast genome has relatively simple organization with ~6000 open reading frames (ORFs) that are well-defined and are mostly devoid of introns. The actual information about the yeast genome, gene annotation and function is presented in the Saccharomyces Genome Database (SGD; www.yeastgenome.org, accessed on 28 June 2023) [3,4]. The SGD represents a search and analysis tool to explore yeast genome data and allows sequence analysis with an incorporated genome browser and BLAST. It also enables functional gene analysis such as gene ontology, gene expression and interaction analysis as well as many other options.

**Figure 1:**
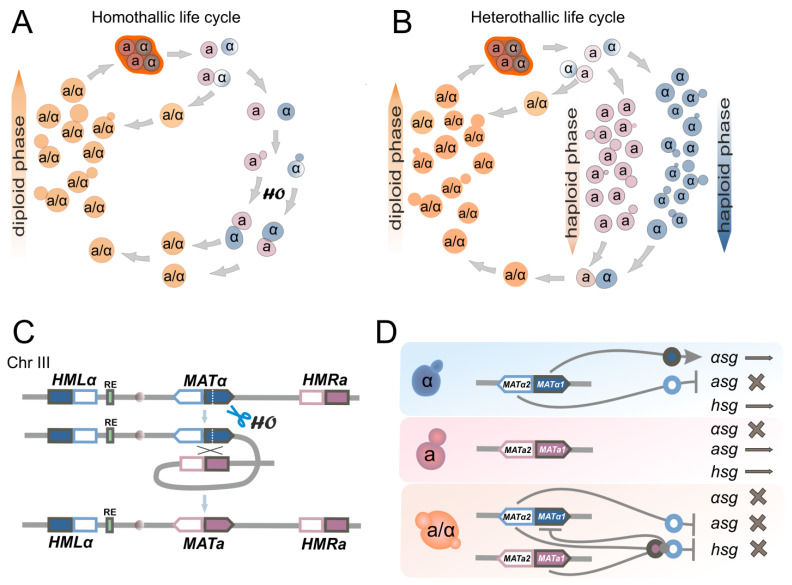
Life cycle and genetic control of the mating type in yeast *S. cerevisiae*. (**A**) Life cycle of homothallic (**A**) and heterothallic (**B**) strains of yeast *S. cerevisiae*; (**C**) Relative location of the mating-type genes in chromosome III, RE—recombination enhancer, HO—*MAT* specific HO endonuclease. Recombination between silent cassette and *MAT* locus results in mating-type switch; (**D**) Regulation of transcription of the mating-type specific genes in haploid and diploid cells of yeast *S. cerevisiae* (a simplified scheme). asg—a-specific genes, αsg—α-specific genes, hsg—haploid-specific genes.

*S. cerevisiae* has a genome that can easily be manipulated; that is an advantage for the construction of heavily modified strains carrying multiple different modifications. Yeasts can exist in both haploid and diploid states during their life cycle. In the haploid state a yeast cell contains 16 chromosomes. During mating, haploid cells can fuse to form diploid cells that can undergo meiosis (a process called sporulation in yeast) to generate four haploid spores. This has been extensively used for genetic (tetrad) analysis in yeast. There are two mating types in haploid *S. cerevisiae*—a and α, which are controlled by the *MAT* locus mapped on the right arm of chromosome III [5]. Yeasts can be homo- and heterothallic, meaning that they can switch their mating type easily and undergo mating with diploidization (homothallic) or have a relatively stable mating type and exist as haploids through many generations (heterothallic) (Figure 1A,B). Homothallic yeasts exist in a diploid state for most of their life cycle. Laboratory yeast strains are usually heterothallic because they lack the HO endonuclease, which is involved in mating-type switching by introducing a break into the *MAT* locus [6] (Figure 1C,D, see Section 7 for more details). Spontaneous changes of ploidy in laboratory yeast strains are not rare and occur with a rate about 10^−5^–10^−4^ (autodiploidization through whole-genome duplication) or lower (~10^−5^–10^−7^) if it involves mating-type switching and hybridization [7,8].

Types of yeast genome modification used for the construction of laboratory and industrial strains include point mutations, gene deletion, gene disruption, gene or expression cassette insertion, aneuploidy, polyploidy, introduction of directed DSB, mating-type switch and others. It should be noted that almost any manipulation with non-essential genes can be carried out in yeast. The function of the essential genes can be studied through manipulation with diploid yeast with subsequent tetrad analysis or through studying the alleles which can transiently inactivate gene function under certain conditions, the so-called conditional alleles. One should keep in mind that the *S. cerevisiae* genome is very compact (~12 Mb) with relatively small intergenic regions. Such a peculiarity can complicate the design of gene knock-outs because removal of the entire open reading frame or its significant part can affect regulatory sequences of the adjacent genes. Sequences inside the cassette may also be a cause of adjacent genes’ dysregulation [9]. It may therefore be recommended to confirm the effect of a genetic manipulation by several different approaches in cases where it might be important.

The relative simplicity of yeast genome modification is based on the combination of three factors: the high efficiency of homologous recombination, different transformation methods, and the availability of various selective markers.

## 2. Homologous Recombination and Introduction of Directed Double-Stranded Breaks

**Figure 2:**
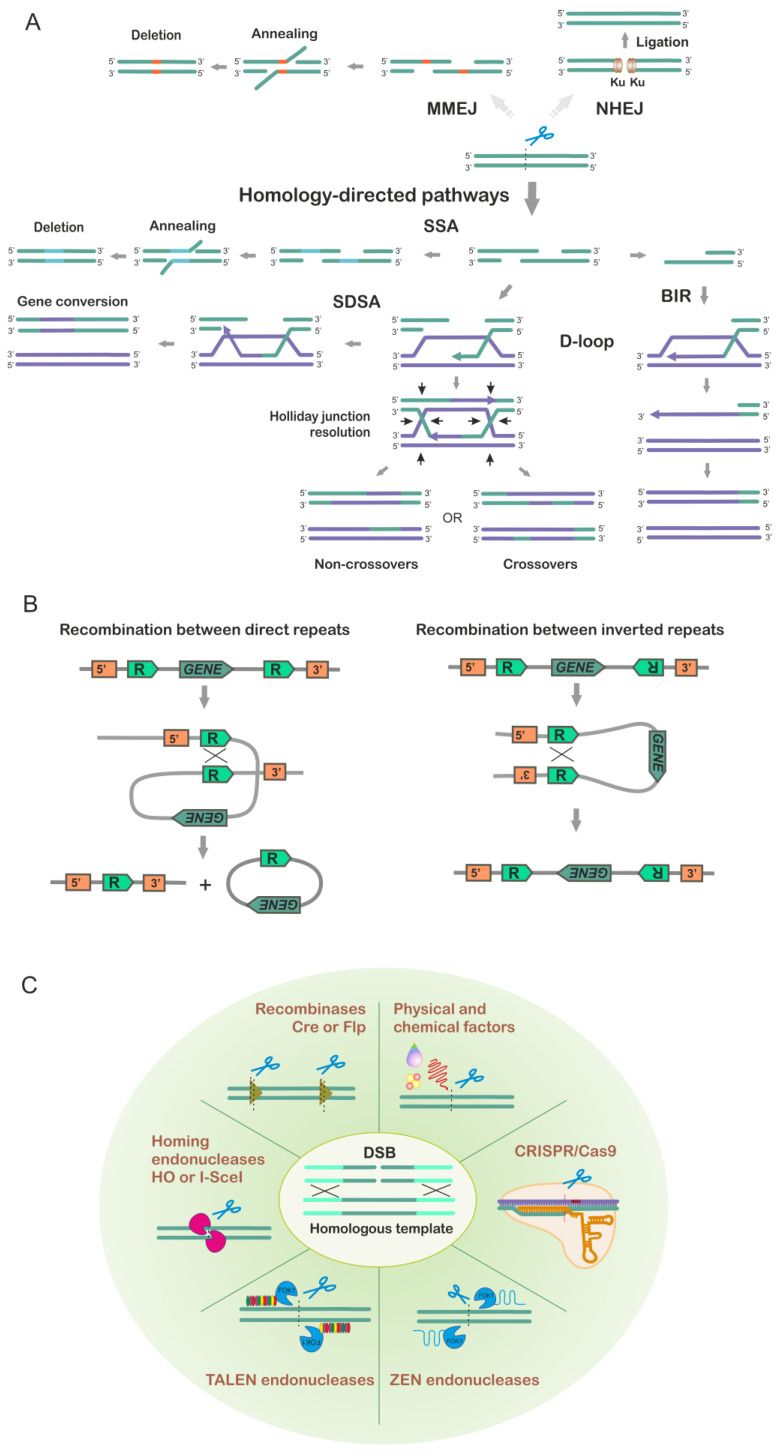
Mechanisms of DSB generation and repair and their outcome. (**A**) Mechanisms of homology-directed and non-homologous DSB repair, NHEJ—non-homologous end joining, MMEJ—microhomology-mediated end joining, BIR—break-induced replication, SSA—single-strand annealing, SDSA—synthesis-dependent strand annealing, a double Holliday junction and an example of its resolution are shown; (**B**) Recombination between direct and inverted DNA repeats; (**C**) Mechanisms of DSB generation in yeast cells.

Homologous recombination (HR) and non-homologous end joining (NHEJ) are the two classic mechanisms allowing the repair of DNA double-stranded breaks (DSBs) which are the most severe types of damage to the cell [10]. While NHEJ simply ligates the broken DNA ends together without referencing to the information that might be lost at the site of the DNA damage, HR seeks a template with homology to the DSB site and uses it to recover the information (Figure 2A). Not surprisingly, the homology-directed DNA repair (HDR) system plays a fundamental role in the stability of the genome. This system enables accurate repair of DSBs and also helps to repair stalled replication forks, the events that may happen at the sites where DNA sequence is damaged (e.g., by intermolecular cross-links or DNA adducts) or compromised by secondary DNA structures [11,12]. HR is extremely important when cells are actively dividing or are exposed to DNA damaging agents. DSBs are potent inducers of HR and this mechanism is initiated by resection of the DNA 5′ ends at the DSBs followed by invasion of the resulting 3′ protruding end into the homologous duplex and formation of a so-called displacement loop (D-loop) [13,14,15]. The D-loop is a substrate for DNA replication machinery which can extend the 3′ prime end of the heteroduplex and restore the disrupted information by DNA synthesis on the new template. The next steps can be different depending on the downstream pathway of HR (Figure 2A). If both ends of the DSB are available for template exchange or strand invasion with the donor duplex, then the HR can be channeled to a double Holliday junction (dHJ) pathway. If the invaded strand after DNA synthesis reanneals to the other resected DSB end and provides a template for DNA synthesis of the second strand, then the pathway is called synthesis-dependent strand annealing (SDSA). This pathway usually leads to gene conversion. If only one end of the DSB is being repaired and D-loop extension proceeds via a migrating bubble with asynchronous leading- and lagging-strand synthesis then the pathway is categorized as break-induced replication (BIR) [16,17,18,19]. In addition, there is one more pathway which is called single-strand annealing (SSA), which is often classified as an HR pathway (Figure 2A). This pathway does not involve D-loop formation and depends on sequence homology between single-stranded DSB ends. In the case of homology, the 3′-protruding ends can anneal to each other forming a duplex; the protruding ends will then be clipped off and the gaps filled in by DNA synthesis. This pathway is related to microhomology-mediated end joining (MMEJ) [20].

Yeast *S. cerevisiae* is characterized by highly efficient HDR while its NHEJ system is far less effective. This fact is the basis of the easiness that is characteristic of yeast genome manipulations. In various approaches and methods for yeast genome modification, HR is an obligatory mechanism for inserting DNA fragments into the target locus. Yeast cells are usually transformed with a linear donor DNA molecule (a PCR product or a linearized plasmid); the ends serve as a substrate for HR, or alternatively, a DSB may be specifically induced at the target site in the genome. The donor DNA fragment must have homology with the target locus to allow for precise recombination. Once the DSB is created in the genome, the cell’s repair machinery is activated, and the integrity of the DNA molecule is restored by inserting the donor fragment. It should be noted that besides true homologous integration, yeasts can insert different sequences into their genome through a so-called illegitimate recombination which is mediated by microhomology. In fact, even 4 bp sequence microhomology can be sufficient for the integration of an extrachromosomal sequence into the yeast genome [21,22]. The repair of DSB can occur between divergent sequences; thus even when every sixth base pair was mismatched between donor and target sequences, the repair was still detectable at a rate of about 5% of the fully homologous sequences [23]. Another issue is that HR repair of DSBs is not error-free; in fact it generates about 1000 times more mutations than regular DNA replication [20]. In addition, the HR machinery is unable to differentiate between the repetitive sequences in the genome, which can lead to a variety of genome structural changes. Repetitive sequences are frequently used in yeast genome modification for selective marker removals or genome restructuring. Note that the outcome of recombination between the direct and inverted DNA repeats is different (Figure 2B).

Induction of DNA double-strand breaks is often used for scientific purposes, in particular to study DNA repair mechanisms or as part of editing technologies, when DSBs at specific locations in the genome target and stimulate desired genetic changes. There are several methods for introducing DSBs into the genome, including physical methods such as ionizing radiation or ultraviolet light, chemical methods such as treatment with DNA-damaging agents like bleomycin and molecular methods such as the use of endonucleases (Figure 2C). While non-specific DNA breaks can be induced by physical and chemical methods, endonucleases can generate sequence-specific DNA breaks. For genome modification in yeast, among endonucleases the most frequently used are homing endonucleases (HO, I-SceI, I-AniI), and CRISPR/Cas9. Homing endonucleases are enzymes that can recognize and cut specific DNA sequences, allowing for precise editing of the genome. CRISPR/Cas9 is a more versatile system that uses RNA molecules to guide the Cas9 enzyme to specific DNA sequences, where it can make targeted cuts. Specific DSBs can also be induced in the yeast genome using custom-engineered zinc finger nucleases (ZFNs) and transcription activator-like effector nucleases (TALENs). ZFNs consist of a DNA-binding domain derived from zinc finger proteins and a nuclease domain derived from the FokI endonuclease. TALENs are similar to ZFNs in that they also consist of a DNA-binding domain and a nuclease domain. However, the DNA-binding domain of TALENs is derived from transcription activator-like effectors (TALEs), which are bacterial proteins that bind to specific DNA sequences. The DNA-binding domains of ZFNs and TALENs can be engineered to recognize specific DNA sequences, allowing precise targeting of nuclease activity to desired locations in the genome. Once ZFNs or TALENs bind to their target site, the nuclease domain cleaves the DNA, creating double-stranded breaks that can be repaired by HR or NHEJ. Both ZFNs and TALENs have been widely used in genetic engineering of various organisms, including the yeast *S. cerevisiae* [24,25,26,27]. However, in recent years, ZFNs and TALENs have been replaced by CRISPR/Cas technologies due to lower labor costs and greater reliability.

## 3. Yeast Transformation

Genetic transformation is a way for the uptake of exogenous genetic material and its processing in the host cell; thus, it can affect the host cell properties. Different genetic transformation techniques have been developed for *S. cerevisiae* over more than four decades since the first transformation of a laboratory yeast strain [28], including various enzymatic, chemical and physical methods [29,30]. Yeast has a prominent cell wall that usually needs to be removed enzymatically (e.g., by lyticase or zymolyase) or pretreated by chemicals (e.g., polyethylene glycol (PEG) and lithium salts) to allow DNA uptake. The most commonly used method for yeast transformation, combining simplicity, versatility and efficiency, that can be used in almost any setting is the lithium acetate/single-stranded carrier DNA/PEG method [31]. This method is relatively fast and does not require laborious preparation or specific equipment, and we can recommend it as a routine procedure. It is suitable when high-efficiency transformation is required [32] and can be efficiently scaled up [33].

Yeast transformation can also be achieved by various physical methods, reviewed in [34]. Electroporation is one of the most widely used physical methods. It is relatively simple since all it may require, besides an electroporator, is just a brief cell pre-wash in a low conductivity buffer [35,36]. In some cases, PEG may be added or cells may be pre-treated with lithium acetate and DTT [37,38]. Electroporation can even be used for quick transformation of a stationary-phase yeast culture with acceptable efficiency [39]. Recently, scanning electrochemical microscopy was used for localized electroporation of living yeast cells [40].

Interestingly, yeast can be also transformed with T-DNA of *Agrobacterium tumefaciens* [41,42,43]. T-DNA lacks homology with the *S. cerevisiae* genome and integrates at random positions via illegitimate recombination [42]. Deletion of the *ADA2* yeast gene, encoding a component of the ADA/SAGA histone acetyltransferase complexes, increased the efficiency of *Agrobacterium*-mediated transformation [44].

## 4. Selectable Markers Used for *S. cerevisiae* Transformation and Transformant Selection

*S. cerevisiae* allows the use of an extensive set of markers for the selection of transformants both when introducing replicating plasmids or introducing modifications into chromosomal DNA [45]. Most genetic experiments typically use a standard set of selectable prototrophic markers, including *URA3*, *LEU2*, *TRP1*, *HIS3*, and less commonly other markers such as *LYS2*, *ADE1*, *ADE2*, and *MET15*. The selection of transformants/integrants is carried out by compensation of the corresponding genomic mutations of auxotrophy for amino acids or nitrogenous bases in the recipient strains with corresponding prototrophic donor sequences. In the case of the strains that do not carry the desired marker mutations in the genome, as well as in cases where available markers have already been used for the selection of transformants or integrants, dominant markers of antibiotic resistance or other agents toxic to yeast can be used. These markers include *kan^r^* and its derivatives (resistance to G418), *nat* (to nourseothricin), *hph* (to hygromycin B), *cat* (to chloramphenicol), *CUP1* (to increased concentrations of copper ions), *SFA1* (to formaldehyde) and *SMR1* (to sulfometuron methyl) [45,46]. The use of such markers, however, may be sometimes limited due to the fact that many of the antibiotics and chemical agents used for transformant/integrant selection are expensive and/or have a strong effect on the physiology of yeast cells; some of them, in addition, are toxic to humans. A promising direction may be the usage of genes from other organisms as selective markers which allow yeasts to grow on media with substrates that they do not normally utilize. Examples of such markers are the acetamidase gene of *Aspergillus nidulans*, which allows yeast to grow on media with acetamide as the only source of nitrogen [46], the phosphite dehydrogenase gene of the bacterium *Ralstonia*, which allows the growth on media with phosphites as the only source of phosphorus [47], and *Kluyveromyces lactis LAC4*/*LAC12* and *Lipomyces starkeyi LSD1* genes, which provide growth on media with lactose and dextran as the sole carbon sources, respectively [48,49].

A great advantage of the *Saccharomyces* yeast is the availability of a number of counterselectable markers, which allow selecting for the absence of their functional allele in a cell. Using specific media, it is possible to select mutants for such a gene, clones that have lost a plasmid with a given marker gene or have excised a cassette with such a gene after its integration into the chromosome. The most widely used counterselectable marker is *URA3*, which does not allow for growth in the presence of 5-fluoroorotic acid (5-FOA) [50,51]. Despite its wide usage one should keep in mind the mutagenic potential of 5-FOA. By the action of the *URA3* encoded orotine-5’-monophosphate (OMP) decarboxylase 5-FOA is converted in the cell into a toxic 5’-fluorouridine monophosphate that disrupts the pyrimidine nucleotide pool balance. 5-FOA was reported to induce chromosome alterations in prototrophic *Candida albicans* [52].

Among other counterselectable markers it is worth mentioning *TRP1*, *LYS2* and *CAN1*, which can be counterselected on the media with 5-fluoroanthranilic acid, α-aminoadipate and L-canavanine, correspondingly [53,54,55]. An example of a heterologous counterselectable marker is the acetamidase gene of *A. nidulans,* selection for the absence of which can be carried out on the media with fluoroacetamide [46]. Some other useful counterselectable markers and selective conditions are mentioned elsewhere [45]. Additionally, useful vectors for manipulations in yeast and bacteria are reviewed in [56].

## 5. Classical Approaches to Genome Modifications in *S. cerevisiae*

### 5.1. One-Step Gene Disruption and Deletion Methods

Gene disruption is one of the common methods for gene inactivation (knock-out). This method implies the insertion of a selectable marker into the coding sequence of the gene leading to its interruption.

**Figure 3:**
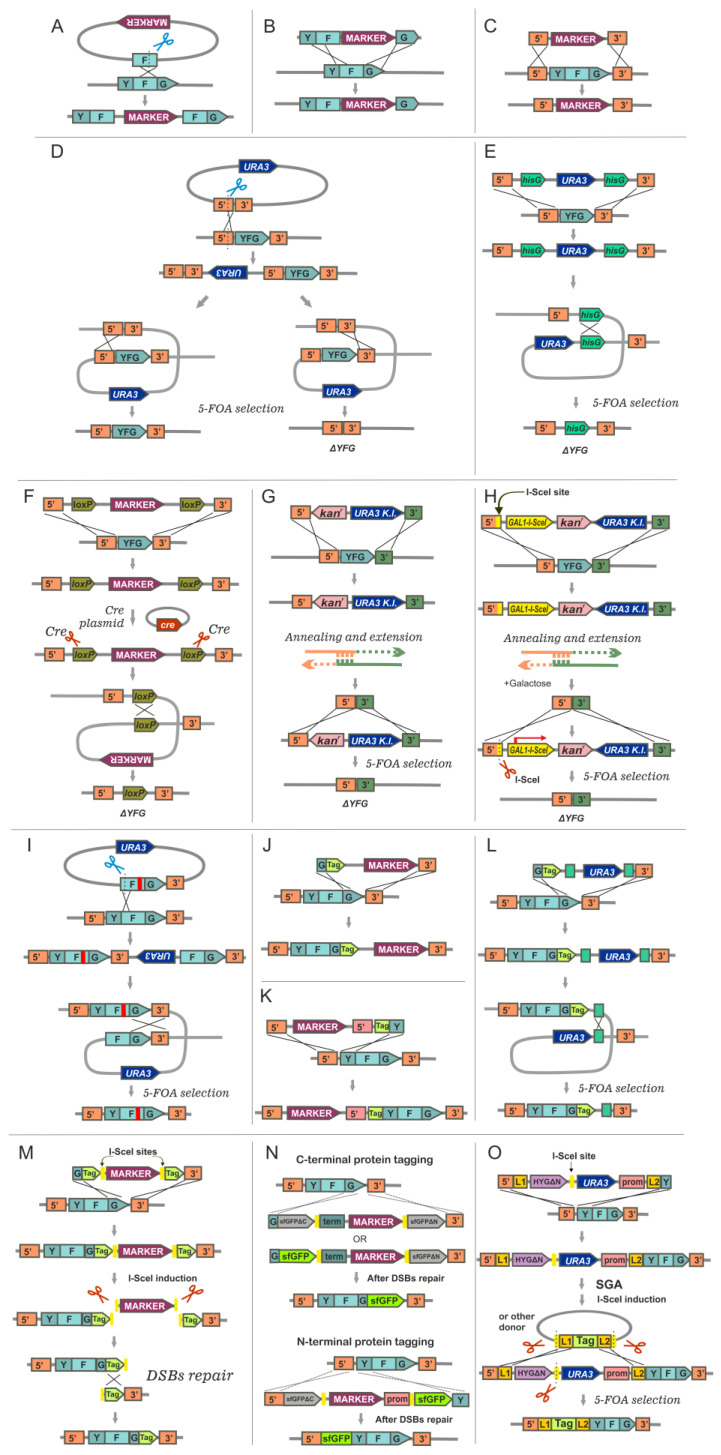
Classical approaches to genome modifications in *S. cerevisiae* based on homologous recombination: (**A**) one-step gene disruption using a plasmid with an internal fragment of the target gene and selectable marker; (**B**) one-step gene disruption using a fragment consisting of the target gene with insertion of a selectable marker; (**C**) one-step gene deletion using a selectable marker flanked with sequences homologous to 5′ and 3′ non-coding regions of the target gene; note that the homologous sequences can be PCR-generated and be as short as 40–50 nucleotides for effective integration; (**D**) two-step gene deletion (pop-in/pop-out method); (**E**) two-step gene deletion using a cassette with *hisG* repeats; (**F**) two-step gene deletion using the Cre/*loxP* system; (**G**) an example of the *delitto perfetto* approach for precise gene deletion; the orientation of the selectable markers can be different in different variants of this system; (**H**) the break-mediated *delitto perfetto* approach for gene deletion; the orientation of markers can be different in different variants of this system; note that both (**G**) and (**H**) approaches can be used for gene disruption with consequent gene deletion; they also allow introduction of point mutations (see [62] for more details); (**I**) two-step method for introduction of point mutations. At the second step the most probable way of the plasmid sequence excision is specified, which results in the mutant allele of the target gene in the chromosome; (**J**) one-step insertion of the C-terminal tag into a chromosomal copy of the target gene; (**K**) one-step insertion of the N-terminal tag into chromosomal copy of the target gene; (**L**) insertion of the C-terminal tag into a chromosomal copy of the target gene. After insertion a selectable marker can be excised due to recombination between direct DNA repeats (e.g., *hisG* repeats); (**M**) seamless gene tagging by the endonuclease-driven HR approach as it was proposed in [63]; (**N**) examples of seamless gene tagging with the superfolder green fluorescent protein (sfGFP) cassettes, see for details [63]; (**O**) SWAp-Tag (SWAT) strategy for easy module exchange [64]. YFG (Your Favorite Gene)—gene to be deleted, inactivated or modified. Recombination events between homologous regions are shown. Blue scissors denote plasmid linearization by restriction endonuclease at a unique site, red scissors denote endonuclease expressed in the cell. (**C**,**E**–**H**,**J**–**O)**—illustrate PCR-based approaches for cassette generation.

One can disrupt a gene using an integrative plasmid containing an internal fragment of the target gene and a yeast selectable marker (Figure 3A). Transformation of a recipient strain with this plasmid, linearized at a site within the target gene fragment, results in plasmid integration to form two truncated gene derivates in the chromosome [57]. This method is very simple and effective. However, its main disadvantage is the instability of the resulting structure. Due to recombination between direct repeats generated after plasmid integration, the plasmid sequence can be cleaved off from the chromosome, and as a result the wild-type gene sequence is restored. The frequency of such events is high enough (about 10^−4^) to complicate its usage for the construction of stable strains. However, this method can be recommended as an express approach to test for the phenotype of the target gene inactivation.

The classic approach to gene disruption is the one-step disruption method which is based on the fact that linear DNA fragments carrying a selectable marker gene flanked by regions homologous to the yeast target gene integrate at the corresponding chromosomal locus by homologous recombination with high efficiency [58,59,60]. To use this method, it is necessary to obtain a plasmid carrying the target gene, into which the marker gene is inserted. A plasmid fragment consisting of a disrupted target gene is used to transform the recipient strain (Figure 3B).

Insertion of the fragment into the chromosome due to homologous recombination leads to the substitution of the target gene in the chromosome for its disrupted version from the plasmid fragment. Usually, this approach leads to target gene inactivation; however, truncated version(s) of the affected gene can sometime be expressed, which can sometimes lead to the presence of residual activity of the encoded protein. To overcome this problem, it is better to insert the marker gene, whenever possible, into the 5′ terminal part of the coding sequence rather than into its 3′ part. A frequently used modification of this approach involves insertion of the selectable marker gene instead of the entire target gene’s coding sequence or its portion (sometime called disruption with deletion), thus allowing a strict knocking out of the target gene (Figure 3C).

The fact that the flanking homology regions can be as short as 30 base pairs enabled the development of the now widespread methods for targeted sequence integration into the yeast genome that commonly utilize a special cassette containing a selectable marker flanked with PCR-introduced short sequences homologous to 5′ and 3′ flanking regions of the target sequence in the genome [61]. A whole target gene or any of its parts can be substituted in such a way for the sequence from the cassette (Figure 3C). The selectable cassette is amplified using hybrid primers in which the 3’ portion (usually 18–20 bp) is homologous to the flanking regions of the selectable marker in a cassette and the 5’ portion (usually 40–50 bp long) is homologous to sequences flanking the gene or region to be deleted or modified.

After transformation with the PCR product, homologous recombination at short homology regions leads to the replacement of the target gene or the genomic region of choice with a selectable marker [65,66]. In rare cases the efficient recombination between the target sequence in the genome and a cassette carrying short homologous flanks can be impeded due to the chromatin structure or other obstacles [14]. In such cases longer regions of homology of about 90–100 bp to the target gene can be helpful to achieve the integration of the cassette. One should note that the usage of short homology regions for integration is effective when the selectable marker does not have an extended homology with the genome sequences. This is usually achieved when the corresponding auxotrophic allele which is being compensated represents a deletion of a large portion of a gene. At the same time auxotrophic alleles represented by point mutations (e.g., nonsense mutations) carry the entire sequence homologous to the marker gene in the cassette, which often results in the integration of the processed cassette into the genomic position of the selectable marker locus and not into the target locus.

Currently, there is a wide choice of plasmids containing various cassettes for yeast genome modification, while the cassette can contain from one to three marker genes. Heterologous genes such as *K. lactis URA3*, *his5+* of *Schizosaccharomyces pombe* homologous to *S. cerevisiae HIS3*, or the *kan^r^* gene from the *Escherichia coli* Tn903 transposon determining the resistance of yeast to the antibiotic G418 are favored for the disruption and deletion cassettes. Usage of such markers reduces possible recombination between the marker gene and sequences in the yeast genome, thus maximizing the involvement of the short flanking segments at both ends of the cassette in HR with chromosomal sequences [60,67,68,69,70,71].

The first heterologous, dominant disruption marker was the *kan^r^* gene providing resistance to G418 [68,72]. A series of pFA plasmids carrying different modifications of the kanMX cassette (such as kanMX2, kanMX3, kanMX4, kanMX6) was created. The *kan^r^* ORF was fused to the transcriptional and translational control sequences of the *TEF* gene of the filamentous fungus *Ashbya gossypii* in these modules [68,69].

The kanMX4 cassette containing *kan^r^* has been used to generate yeast gene knockout collections (YKO) of almost 6000 genes, where each of the strains carried a deletion of a particular gene (deletions of essential genes were obtained in diploids) [73,74], reviewed in [75]. YKO collection strains may be used for knocking-out of a particular gene in any yeast strain. To do this, one can simply PCR-amplify the kanMX cassette with the flanking regions (100 bp or more) from the genomic DNA of the corresponding YKO strain, transform the recipient strain with the PCR product and select G418-resistant transformants. This procedure should be more effective compared to conventional PCR-based disruption due to the presence of longer regions for homologous recombination.

It should be noted though that usage of the kanMX cassette for gene knockouts was reported to be associated with significant alterations in the expression of the adjacent genes at the transcription and translation level; thus the kanMX cassette affected translation of the neighboring genes in about 10–20% of knockout strains in the yeast deletion library [9]. Some of the heterologous cassettes may carry identical or homologous sequences to each other. For instance, this concerns the widely used and very convenient drug-resistance MX series based on kanMX3 and kanMX4 cassettes. They were constructed by replacing the *kan^r^* ORF with either the *pat*, *nat1* or *hph* open reading frames while the rest portion of the cassettes (*TEF* promoter, *TEF* terminator) remained the same [71]. This may be important when making sequential knock-outs using several MX cassettes since it may lead to integration of the second MX cassette into the MX cassette inserted in the first round of genetic modification (cassette mis-targeting). The easiest way to select against such events is to select integrants on media containing antibiotics for both drug-resistance markers.

In all cases transformants carrying the correctly integrated disruption or deletion cassette may be identified by diagnostic PCR assays using primers complementary to sequences located within the cassette and primers flanking the sites of integration. A PCR product of the expected size from any side of the cassette will be obtained only if the deletion cassette has been correctly inserted into the genome by homologous recombination. Alternatively, one can amplify the whole cassette from external (outside the area of integration) primers and verify its size (this only works if the expected size after integration is different from the intact sequence). This PCR product can be sequenced to further verify the correct integration.

### 5.2. Two-Step Gene Deletion Methods

Regardless of the specific method of gene deletion or disruption, this genome modification results in the introduction of a selectable marker gene into the yeast genome. Sometimes this can create a problem, for example, if more than one gene needs to be inactivated in one strain and the number of markers suitable for this strain is limited. Multiple gene deletions can be carried out using the approaches that support removal of the unwanted cassette from the genome after its insertion by mitotic or recombinase-mediated recombination, thus allowing the same selectable marker to be reused for the next round of genome modification. Most of these methods rely on the usage of counterselectable markers, such as *URA3*.

The classic approach to create a gene deletion in the yeast chromosome is two-step integration/excision (pop-in/pop-out) procedure (Figure 3D) [59,76]. To achieve this, an integrative plasmid carrying the selectable marker and the sequence corresponding to the target region for integration with required modifications is constructed. For instance, a sequence carrying 5′ and 3′ flanking regions without a gene coding sequence should be used for the whole gene deletion. At the first step, the recipient strain is transformed with this plasmid linearized at the site chosen for recombination (Figure 3D). As a result of plasmid integration in the chromosome, a duplication is formed containing the wild-type gene copy and its altered variant (e.g., deletion) and the plasmid sequences in between. At the second step, excision of the plasmid sequence containing the selectable marker occurs, which may result in the generation of the altered sequence of the target locus in the chromosome or in the restoration of its intact sequence (Figure 3D). Which of the two options is implemented depends on whether recombination between the duplicated regions occurs upstream or downstream of the altered sequence. The integration (pop-in) and excision (pop-out) events must occur on different flanking sequences for the alteration to be retained after pop-out. Clones with excision of the plasmid sequence should be selected on specific media allowing counterselection (e.g., 5-FOA media for the *URA3* marker) and then screened for the presence of the introduced gene modification. If 5’ and 3’ flanking sequences of the target sequence differ significantly in length, the restriction site for plasmid linearization should be in the shortest flanking sequence to increase the yield (frequency) of clones with the deletion [59].

An alternative to this method is to use specially designed cassettes that support recombination between the cassette sequences and thus allow excision of the marker. One example is a set of cassettes comprising *URA3* flanked with direct repeats of heterologous sequences, such as the *Salmonella typhimurium hisG* gene (Figure 3E). After the insertion of the cassette, clones can be selected on the medium with 5-FOA to select *URA3* excision events occurring due to recombination between the direct *hisG* repeats [77,78]. Another approach takes advantage of the *loxP* direct repeats and Cre recombinase of bacteriophage P1 [79]. The marker gene is excised after the cassette insertion via recombination induced by the Cre recombinase (Figure 3F). Since Cre-induced site-specific recombination is very effective, there is no need to use counterselectable markers. A number of cassettes carrying different *loxP* flanked marker genes are currently available [67,80,81,82,83]. The Flp/FRT method taking advantage of the Flp recombinase has been used for conditional gene deletion when a targeted FRT flanked gene can be excised after the induction of Flp expression [84]. It should be noted that the usage of direct repeats for excision of the cassette sequences may result in region mis-targeting in multiple gene-deletion settings. A single genomic copy of *hisG* decreases the percentage of integration at the target locus from 44% to 4.5% and two genomic *hisG* copies decrease it to less than 1% [85]. Two or more *loxP* sites in the genome remnant after genetic manipulations may provoke chromosomal rearrangements [81,86]. Cassettes with mutated *loxP* sites which are incompatible with the standard *loxP* sites might be a good solution for consequent gene deletions using Cre/*loxP* system [82]. 

A method allowing the creation of seamless deletions in the yeast genome, the *delitto perfetto,* was introduced about 20 years ago [87,88]. The method was based on PCR-amplification of a special cassette and oligonucleotide genome targeting that provided direct selection for different chromosomal modifications including gene deletions. The name ascribed to this versatile and accurate method was derived from the idiomatic Italian term for perfect murder since there was complete elimination (i.e., perfect deletion) of the marker sequences that were used for selection. The *delitto perfetto* procedure for gene deletion is accomplished in two steps, both of which involve homologous recombination (Figure 3G) [88]. The first step involves integration of a COunterselectable REporter (CORE) cassette into a desired genomic locus. A CORE cassette usually contains two (or more) markers one of which is a reporter (e.g., *kan^r^*) and another is counterselectable (e.g., *K. lactis URA3*). A cassette is PCR-amplified using hybrid primers: the 5’-part of which (40–50 bp) is complementary to the flanking sequences of a gene to be deleted and the 3′-part is complementary to the cassette flanks. The cassette integration is accompanied by a deletion of the target gene. The second step is accomplished by transformation of the strain obtained at the first step with the appropriate targeting oligonucleotides (90–140 bp) having homology to sequences which flank the CORE cassette in the chromosome. As a result, the CORE cassette is excised from the chromosome, which results in a precise deletion of the target sequence (Figure 3G). Either single-stranded or double-stranded oligonucleotides can be used at the second step, although double-stranded oligonucleotides are more effective [62]. A modification of the *delitto perfetto* approach, known as break-mediated *delitto perfetto*, took advantage of the HR repair of double-strand breaks in yeast (Figure 3H) [88]. In this modification, the CORE cassette includes additional galactose-induced gene coding for the I-SceI endonuclease, and the I-SceI recognition site (usually introduced with oligonucleotides during amplification of the cassette), which is not found anywhere else in the yeast genome. The I-SceI endonuclease generates the DSB in the target locus, which increases oligonucleotide recombination more than 1000-fold, reaching efficiencies as high as 20% of all cells [62]. It should be noted that the resultant cassette is large enough for routine amplification. Its size can also interfere with transformation/integration into the genome. Functional equivalents of *delitto perfetto* that can satisfy different needs are available. One of them, MX4blaster, represents an MX4-compatible cassette that can facilitate scarless kanMX4 removal [89].

All two-step gene deletion methods imply a two-step verification procedure. First, the cassette integration is verified by PCR similar to the one-step procedure. Next, the targeted region is amplified from external primers after the second step (cassette or marker excision). The removal of these sequences can be monitored by the size of the PCR product.

### 5.3. Gene Modifications Methods

The pop-in/pop-out method can be used for the introduction of different types of gene modifications, including point mutations, small indels, gene truncations, etc. (Figure 3I). A corresponding modified allele (a complete gene sequence or its part) is introduced into the genome within the integrative cassette and is expected to be retained in the genome after cassette pop-out with a certain frequency. PCR-based gene modification approaches can also be helpful in this setting if a sequence corresponding to a modified allele is used for the second round of transformation and HR. For instance, the *delitto perfetto* can be effectively used for the introduction of point mutations [62]

PCR-based gene modification approaches can be also used for alteration of the gene expression pattern by placing the target gene under the control of a new promoter or to obtain a hybrid gene in the chromosome that encodes a protein labeled with N-terminal or C-terminal tags for fluorescent or immunochemical analysis (Figure 3J,K). In this case, the primers for the cassette amplification are designed in such a way that homologous recombination leads to the insertion of a promoter or tag tightly linked to a selective marker into the chromosome at the appropriate position adjacent to the 5′ or 3′ end of the gene coding sequence. When a gene is placed under the control of a new promoter, the native promoter is usually replaced with a new one. It is worth mentioning that this approach was applied when creating a collection of about 800 strains in which particular essential genes were put under the control of a tetracycline-repressible heterologous promoter, which allowed for the study of terminal phenotypes in the case of sublethal reduction in the level of transcription of these genes. This approach allowed for the establishment of functions of some essential genes with unknown functions [90]. There is currently a large set of plasmids with cassettes that allow such N- and C-terminal modifications of target genes [69,70,91,92,93,94,95]. Some cassettes allow excision of the selectable marker, inserted along with the corresponding tag (Figure 3L). A promising two-step method was developed allowing seamless N- and C-terminal tagging of genes. At the first step, a PCR-generated module consisting of two direct repeats of the tag sequence and a selective marker in between is targeted to the desired locus by HR. A module contains two sites for I-SceI endonuclease flanking selective-marker sequence (Figure 3M,N). Yeast promoter or terminator sequences may be included in the module which allows for retaining the target gene proper expression at this step. At the second step, I-SceI expression leads to marker excision; the resulting DSB can then be repaired by homologous recombination between the two repeats of the tag sequence. This should effectively remove all auxiliary sequences from the integrated module, leaving a single copy of the tag in the desired site of the genome [63,96]. Such seamless gene tagging allows for the preservation of endogenous gene regulation. This method can be combined with synthetic genetic array (SGA) high-throughput strain construction [97,98,99] for gene function analysis in a minimally-perturbed context [96].

### 5.4. Yeast Libraries

*S. cerevisiae* has been used for the construction of systematic collections of strains in which each strain carries a certain gene with required modifications, all executed in a similar manner. Such strain collections are termed libraries and are frequently used in genome-wide functional studies and different screenings. One example of such a library is the YKO collection discussed in Section 5.1. Libraries of conditional loss-of-function mutations in essential genes were extensively reviewed in [100] and include tetracycline-repressible gene collection, collection of temperature-sensitive alleles, collections of hypomorphic alleles and the DAmP (decreased abundance by mRNA perturbation). Fusion libraries are exceptionally helpful in interactome analysis [101]. Classical library construction is an extremely laborious and expensive procedure, requiring thousands of primers, transformations and validation steps.

Transposon mutagenesis is a powerful tool for the rapid creation of a large set of mutations. Various transposon-insertion libraries for yeast modification have been generated in Michael Snyder’s laboratory [102,103,104,105,106,107,108]. Briefly, the mutagenizing module usually consists of a mini-transposon (mTn) featuring only the terminal ends that are necessary for transposition, a *lacZ* or *GFP* reporter (for detection of in-frame fusions), a marker for selection in bacterial cells and a marker for selection in yeast; additionally the module may contain *lox* recombination sites and an epitope tag. Such a module is used for mutagenesis of the yeast genomic DNA library in vivo in *E. coli*, then the insertional library is transferred into the yeast cells and the resultant mutant alleles are obtained via homologous recombination. The procedure is explicitly described in [102].

A SWAp-Tag (SWAT) method that enables one parental library to be easily modified to another one has been proposed to facilitate the library creation procedure [64]. The SWAT method relies on an acceptor tagging module carrying the *URA3* selectable marker, a truncated version of the hygromycin B resistance marker, a restriction site for the endonuclease I-SceI and short (45 bp) homology arm sequences providing recombination. This tagging module is integrated into the genome and can be easily swapped to another construction upon I-SceI induction (Figure 3O). This is achieved through mating of the constructed library to a donor strain which harbors the I-SceI endonuclease encoding sequence under an inducible *GAL1* promoter and a donor plasmid that contains the desired module to be ‘swapped in’, also flanked by I-SceI sites. After the SGA procedure for mating, sporulation and selection of the haploid spores of choice, the I-SceI expression is induced and DSB generated within the SWAT module facilitates its replacement for a donor sequence that contains corresponding homologous flanking regions. The selection for the new module can be carried out on 5-FOA media; additionally the hygromycin B resistance marker should be restored to the full-length sequence by precise recombination [64]. The method allows different modifications including the tagging of proteins at the N or C terminus, namely, N-SWAT and C-SWAT. Genome-wide libraries with N-SWAT and C-SWAT acceptor modules have been created that allow diverse modifications to study yeast proteome [109,110].

## 6. CRISPR/Cas9 Genome Editing in Yeast

### 6.1. The Advantage of CRISPR/Cas9 and Its Usage for Genome Editing

**Figure 4:**
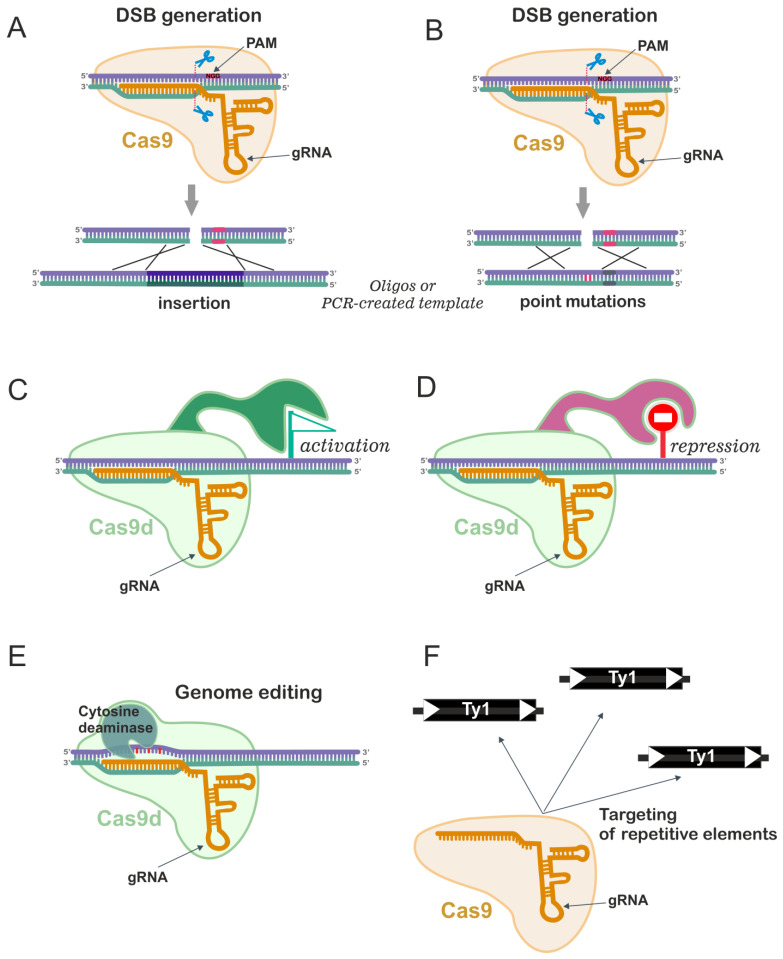
The use of the Cas9/gRNA complex as a guiding tool for delivering various enzymatic activities or regulatory factors to a specific locus in the yeast genome. Induction of DSB at specific site to induce (**A**) insertion or (**B**) point mutation in the target gene; dCas9/gRNA complex lacking the endonuclease activity is able to regulate transcription by targeting (**C**) transcription activator or (**D**) transcription repressor to promoter of the gene of interest; (**E**) dCas9/gRNA complex fused with cytosine deaminase edits DNA by deaminating cytosine to uracil; (**F**) Cas9/gRNA complex targets repetitive elements in the genome to stimulate multiple insertions of the gene of interest in the yeast genome.

**Figure 5:**
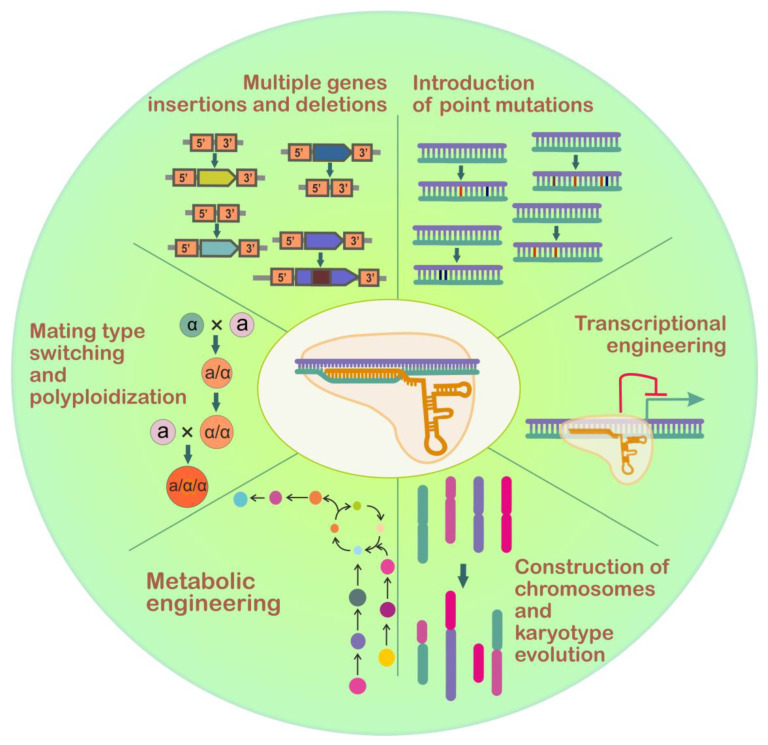
Overview of CRISPR/Cas9 mediated tools used in fundamental and applied research for manipulations with yeast genetic material.

The development of CRISPR/Cas9 systems has allowed researchers to perform highly efficient gene knock-ins and knock-outs without using the selection markers [111]. This system is extremely helpful when multiple genes need to be inactivated or modified. Such tasks are frequent in *S. cerevisiae* metabolic engineering for generation of strains adapted for the production of useful chemicals [112]. Two-step classical methods of yeast genome modification are usually time-consuming. Additionally, copies of repetitive DNA may be retained in the genome if the selectable marker removal has been repeatedly conducted by recombinase-based methods (Cre/*loxP* or Flp/FRT) and these DNA repeats may be a cause of genome instability [113]. At the same time, CRISPR/Cas9 allows fast and simultaneous modification of several genes. One should note though a serious pitfall: the CRISPR/Cas9 complex can recognize target sequences with mismatched bases, thus generating undesirable multiple DSBs off-site [114]. Despite this, CRISPR/Cas9 is widely used for many different applications, some of which are beyond simple gene knock-ins/knock-outs (Figure 4 and Figure 5).

In a standard setting, the bacterial CRISPR system of *Streptococcus pyogenes* requires the Cas9 nuclease and the RNA complex, consisting of the CRISPR RNA (crRNA) required for target recognition and cleavage and the trans-activating CRISPR RNA (tracrRNA), required for the maturation and processing of crRNAs [115,116]. crRNA and tracrRNA can be combined in one chimeric molecule which is often referred to as single guide RNA (gRNA, or sgRNA) since it guides the Cas9 nuclease to a target region. The joint expression of Cas9 and gRNA leads to a DSB at the target DNA site recognized by the crRNA (protospacer) if a protospacer-associated motif (PAM)—a genomic sequence required for cutting—is adjacent to the 3’-end of the protospacer (Figure 4A). In the case of Cas9 from *S. pyogenes* a 5’-NGG-3’ PAM motif is required, but 5′-NAG-3′ can be occasionally recognized. The DNA breaks are then repaired by either NHEJ or HDR: in mammalian cells, Cas9-induced breaks are more often repaired by NHEJ, while in yeast, HDR is more common. In yeast, Cas9-induced breaks are used to stimulate homologous recombination with a donor template DNA (usually, a long oligonucleotide or PCR product) containing alterations in the target locus and a mutated PAM motif to prevent further DNA cleavage [111,117] (Figure 4A,B). Since Cas9-induced DNA breaks prevent the growth of unedited cells containing the original target DNA segment, Cas9 can effectively induce genome editing in yeast without the need for a selectable marker [117]. Various studies have shown that gene disruptions/integrations can be achieved with efficiency values up to 100% [117,118]. Therefore, the use of CRISPR/Cas9 has increased the efficiency of natural recombination methods in yeast, which has contributed to their active involvement in the development of genome-editing technologies. The efficiency of HR between the target region and single-stranded oligonucleotides was increased by five times when Cas9 was used compared to the variant without Cas9, and by 130 times in the presence of double-stranded oligonucleotides [117].

There are two main approaches to CRISPR/Cas9 targeting of the yeast genome: integration of the Cas9 expression cassette into the genome and expression of Cas9 from a plasmid vector. Integration of the Cas9 cassette into the genome has some advantages, such as more stable Cas9 expression and the reduced size of integrative plasmids, thereby increasing transformation efficiency [119]. However, the Cas9 cassette needs to be removed after genome editing. A comprehensive set of tools has been developed by a group of scientists to simplify yeast gene editing using CRISPR/Cas9 [120]. This toolset includes (1) the Yeastriction software tool that can help with gRNA design, minimize off-target activity, and maximize efficiency; (2) a set of the plasmids expressing gRNA, including eight plasmids with a single gRNA and eight plasmids with two gRNAs, including one of nine selectable markers (*S. cerevisiae* or *K. lactis URA3*, *amdSYM*, *hph*, *kan^r^*, *LEU2*, *nat1*, *HIS3*, and *TRP1*); and (3) a collection of various haploid and diploid auxotrophic CEN.PK strains with the Cas9 cassette integrated under the *TEF1* promoter. The authors showed that when using three plasmids, each encoding two gRNAs, six genes can be deleted simultaneously with an efficiency of 65%.

The Cas9 endonuclease and gRNAs can be expressed from one or several plasmids. The main advantage of this approach over Cas9 integration into the genome is the ease of removing the plasmid vector. However, the efficiency of transformation may not be very high when multiple plasmids are transformed simultaneously. Additionally, there may be competition between gRNAs encoding sequences during transcription, which can limit the synthesis of individual gRNA transcripts [121]. The expression vectors pML104 and pML107 have become widely used, each of which simultaneously expresses the Cas9 endonuclease and gRNA. Both vectors contain selective markers *URA3* (pML104) and *LEU2* (pML107) and can be co-transformed with a donor DNA [122]. Plasmid vectors pSNR52, pCRCT, pTAJAK plasmids, and pCEC-red are also commonly used for CRISPR-Cas9 genome editing in yeast. The plasmid vector pSNR52 expresses gRNA under *SNR52* promoter and contains the selective marker *URA3* [117]. pCRCT encodes Cas9, tracrRNA, crRNAs, and the *URA3* selectable marker [123], while pTAJAK plasmids express gRNA and contain different selectable markers [124]. pCEC-red, used for the expression of Cas9 endonuclease and gRNA, contains the marker *mRFP1* (codes for the red fluorescent protein), which is replaced by gRNA when cloned using the Golden Gate Assembly method [125].

Although Cas9 is widely used for yeast genome modifications, there is another Cas protein, Cpf1 (aka Cas12a), that has gained some attention for the purpose of genome editing in yeast [126,127]. Cpf1 belongs to class 2/type V CRISPR bacterial endonucleases and has some distinct features compared to Cas9. For instance, Cpf1 is guided by a single crRNA, pre-crRNA is conveniently processed by Cpf1, and its PAM is T-rich and located at the 5′ end of the protospacer [128]. 

### 6.2. CRISPR/Cas9 for Multiple Gene Disruptions and Deletions

As was noted above, the CRISPR/Cas9 system can overcome a problem of selectable marker removal in multiple gene disruption/deletion settings. This system is efficient enough to be used without selectable markers and can support several genome changes at once. The latter is achieved through the usage of multiple gRNAs (or crRNAs in some settings), which may be expressed from independent promoters or the same promoter. Various systems utilizing CRISPR/Cas9 technology for multiplex genome changes in yeast have been created and are available [121]. An elegant approach was proposed by Ryan et al. who expressed multiple gRNAs from one promoter, separated by self-cleaving ribozyme sequences, necessary for splitting the molecule into individual gRNAs [129]. Bao et al. developed a one-plasmid system that expressed an array of three crRNAs under one promoter separated by spacers and tracrRNA and Cas9 from two other promoters, that allowed simultaneous disruption of three genes with an efficiency of up to 87% [123]. The spacers in the crRNA array in this system mimicked direct repeats of the *S. pyogenes* CRISPR array and were cleaved by the endogenous yeast nucleases, resulting in the expression of multiple individual crRNAs. In another study, Generoso et al. created a set of vectors for the simultaneous expression of Cas9 with one or two different gRNAs (each gRNA expressed separately) to “turn off” three genes in one step [130]. Single-stranded DNA donor templates of 80 nucleotides were used for multiplex deletions [130]. Jakocinas et al. provided a multiplex construct for the expression of up to five gRNAs on one plasmid under the *SNR52* promoter [131]. This system used double-stranded oligonucleotides as templates and allowed the simultaneous deletion of up to five genes in a Cas9-expressing strain with efficiency varying from 50% to 100%. Mans et al. created a technique for the introduction of up to six genetic modifications in a single transformation step with high efficiencies. These modifications include deletions, gene insertions and point mutations [120]. Zhang et al. developed a multiplex CRISPR system with a gRNA-tRNA-array cassette that can simultaneously target the function of eight genes, which is currently the highest multiplex tool in yeast [132]. The drawback of the multiple RNA cassette approach is the decrease of efficiency for gRNA or crRNA with their position relative to the transcription start.

The CRISPR/Cas system has also been used for multiple genome editing in industrial polyploid yeast strains. Zhang et al. used Cas9 to knock out four genes in the ATCC 4124 *S. cerevisiae* strain with an efficiency of 15–60%, resulting in four nutrient-deficient strains [133]. Lian et al. used high-copy number plasmids for the expression of gRNAs for modification of a diploid strain (Ethanol Red) and a triploid strain (ATCC 4124). Four genes were knocked out in one step with an efficiency of 100% [134].

### 6.3. CRISPR/Cas9 for Introduction of Point Mutations

CRISPR/Cas9 is also widely used for the introduction of precise point mutations (changes in a single nucleotide) (Figure 4B). Other changes, such as frameshift mutations or the insertion of a stop codon, can be introduced as an alternative to ORF removal for gene knockout. For instance, co-transformation of gRNA targeting a selectable marker and a double-stranded DNA donor template of 90 bp containing a frameshift mutation and replacement of the PAM sequence with a stop codon led to almost 100% mutagenesis of the target locus [117]. The CRISPR system was successfully used to introduce point mutations simultaneously in the *GET4* and *NAT1* genes with 50% efficiency [120]. Note that generation of additional undesired mutations was registered in this study for one of the target genes.

There are also approaches for base editing in *S. cerevisiae* based on the fusion of cytidine deaminases, such as CDA1, AID or APOBEC1, with Cas9 protein or dead Cas9 (dCas9). In this case, base substitutions occur at a distance of ~15 bp from the PAM motif (e.g., C to G or C to T) [135,136,137] (Figure 4E). Limitations of this method include off-target effects and the formation of indels due to the activity of excision repair of deaminated bases.

Yeast libraries are a good model for systematic biological studies, and the CRISPR/Cas system is a practical tool for creating such libraries. A genome-editing system (CHAnGE) based on CRISPR/Cas9 and HDR which can be used to edit the entire *S. cerevisiae* genome at the nucleotide level has been developed [138]. This method can quickly generate thousands of specific single-nucleotide substitutions in the yeast genome. CHAnGE can also generate single-nucleotide substitutions within individual chromosomes. Based on this system, the authors developed a yeast gene library to increase the production of heterologous proteins.

### 6.4. CRISPR/Cas9 in Transcriptional Engineering

Mutations D10A and H804A affecting the RuvC and HNH domains of the Cas9 protein result in a nuclease-deficient Cas9 protein (dead Cas9—dCas9), which still recognizes the target but it is unable to cleave it (Figure 4C,D). By fusing dCas9 with various types of transcriptional regulators, such as transcriptional inhibitors, activators, or epigenetic modification enzymes, CRISPR/dCas9 can be targeted to specific genome sites, leading to different ways of gene regulation [139,140,141] (Figure 4C,D). Interestingly, CRISPR/dCas9 interference can redefine the transcriptional landscape at targeted loci [142].

Several gene regulation strategies are based on this approach. One example is an activation library based on dCas9, which can recognize target sites in promoter regions and regulate the expression of target genes [143]. The authors identified yeast strains with high-temperature resistance using a library of over 260 gRNAs.

Farzadfard et al. obtained a chimeric protein combining dCas9 with the eukaryotic transcriptional activator VP64 and directed it to the minimal *CYC1* promoter (*pCYC1m*) in *S. cerevisiae* [141]. Another study took advantage of dCas9 coupled to a VP64-p65-Rta (VPR) tripartite activator, which can enable upregulation or downregulation depending on the gRNA position, and a library of 3194 gRNAs targeting 168 genes to screen for targets that enhance the production of 3-hydroxypropionic acid (3-HP) [144,145].

Changes in gene expression and activity can be achieved using CRISPRa/CRISPRi. Vanegas et al. combined Cas9 and dCas9 (or dCas9 with a regulatory domain) into a dynamic tool called CRISPR SWITCH, where the switching mechanism is based on the recombination with the dCas9 encoding sequence after Cas9 introduces a break in its own encoding sequence [146].

Lian et al. tested combinations of Cas9 or Cpf1 proteins from different sources (*S. pyogenes*, *Staphylococcus aureus*, *Streptococcus thermophiles*, and *Lachnospiraceae*), with different activation and inhibition domains. As a result, a tri-functional CRISPR-AID system for regulating *S. cerevisiae* genes was developed, consisting of dLbCpf1-VP (CRISPRa), dSpCas9-RD1152 (CRISPRi), and SaCas9 (CRISPRd) [147]. This system was used for simultaneous transcriptional activation, transcriptional interference, and gene deletion resulting in a five-fold increase in red fluorescent protein, a five-fold inhibition of yellow fluorescent protein, and a 95% deletion of the target gene.

### 6.5. CRISPR/Cas9 in Construction of Yeast Chromosomes

Recently, CRISPR/Cas9 has been used for chromosome construction and genome reorganization. A circular synthetic V chromosome was created using CRISPR technology [148]. Luo et al. successfully merged yeast chromosomes using CRISPR/Cas9 and produced a series of near-isogenic strains containing 2 to 16 chromosomes [149]. Shao et al. effectively removed extra centromeres and telomeres in *S. cerevisiae* using CRISPR/Cas9, and then performed 15 cycles of chromosome fusion through homologous recombination [150]. As a result, the 16 natural haploid chromosomes of *S. cerevisiae* were artificially merged into one chromosome in the yeast strain SY14. In 2019, the CRISPR/Cas9 method was used to induce double-strand breaks in the proximal regions of two telomeres of the linear chromosome SY14, and the ends of the double-strand breaks were connected to a donor DNA fragment through endogenous homologous recombination to form a new strain with a single circular chromosome, named SY15 [151]. A protocol for creating functional chromosome fusions in yeast with CRISPR–Cas9 can be found in [152].

### 6.6. CRISPR/Cas9 in Metabolic Engineering

Yeasts are useful cell factories for the production of various pharmaceuticals and chemicals [153]. Multiple gene deletions, gene insertions or point mutations are often required for the construction of strains used in biotechnological applications [154].

The CRISPR/Cas9 technology is efficient for the removal of unfavorable genes required for metabolic construction. For example, Chin et al. mutated the yeast gene *CAR1*, which encodes arginase, thereby reducing the production of the carcinogenic ethyl carbamate (EC), a byproduct of ethanol fermentation [155]. Liu et al. expressed three gRNAs in one cassette and achieved 95% disruption efficiency to select strains with high ethanol production [156].

Horwitz et al. used the CRISPR system to integrate six DNA fragments containing a total of 11 genes and a length of 24 kb into the *S. cerevisiae* genome, ultimately creating a producent prototype for mucic acid [157]. Several approaches have combined CRISPR/Cas9 with the EasyClone vector toolkit designed for conventional yeast metabolic engineering [158,159]. Thus, Ronda et al. developed CrEdit (CRISPR/Cas9 mediated genome Editing), which combined the efficiency of CRISPR/Cas9 with the convenient genome engineering by EasyClone to integrate genes involved in β-carotene biosynthesis into three *S. cerevisiae* chromosomes, thereby increasing its production [124]. In 2016, Jessop-Fabre et al. using a similar approach developed the EasyClone-Marker Free plasmid toolbox, which can simultaneously insert 1-3 DNA fragments into the *S. cerevisiae* genome without the use of selective markers [118]. Facile simultaneous integration of five heterologous genes was achieved in the “PCR & Go” system where only 40 bp homology flanks obtained by PCR were used. The authors inserted genes of the β-carotene (three genes), zeaxanthin (four genes), and astaxanthin (five genes) biosynthetic pathways [160].

A principally different approach has been proposed by Shi et al. who developed the Delta integration CRISPR/Cas (Di-CRISPR) platform allowing the generation of CRISPR/Cas mediated DSBs at the Ty retrotransposon delta sites in the yeast genome. Coupled with HDR of donor DNA at the sites of DSBs this toolkit can efficiently integrate large biochemical pathways into the multiple genome regions in one step without the usage of selective markers [161,162] (Figure 4F). For instance, the method enabled the integration of 24 kb donor DNA into the yeast genome and the construction of strains that produced (R,R)-2,3-butanediol (BDO) using xylose as a substrate. A similar approach was used for the integration of 25 copies of the BDO gene pathway into the yeast genome [163]. Based on the Delta integration GDi-CRISPR system, exploiting different Cas proteins is another tool for multiplex genome editing [164]. Multicopy integration in Ty1 elements using the CRITGI system which overcomes transcriptional silencing governed by Ty elements by altering the Ty1 chromatin structure allowing transcription of the inserted DNA was reported [165]. It should be noted that CRISPR/Cas9 Ty1 targeting results in about 1000-fold elevation of genome rearrangement events and the types of these events depend on the ploidy [166]. An elevation of mitotic recombination resulting in a loss of heterozygosity (LOH) was also noted in diploid strains [166]. It is therefore expected that strains generated via such methods may carry multiple collateral genome changes. Other sequences such as ribosomal DNA (rDNA) repeats may be used for multiplex integration in yeast [167].

CRISPR/Cas9 can be used to replace regulatory elements to enable heterologous gene expression. Kang et al. described an approach to replace the natural promoter with another using the mCRISTAR platform [168], which combines CRISPR/Cas9 and transformation-associated recombination (TAR) [169]. In four rounds of mCRISTAR, up to 32 promoters can be integrated using four auxotrophic markers [168]. Sasaki et al. simultaneously used CRISPR delta integration with multiple promoter shuffling to select efficient producers of endoglucanase II from *Trichoderma reesei* [170]

## 7. Mating-Type Switching in *S. cerevisiae*

The mating type in yeast is regulated by the *MAT* locus mapped on the right arm of chromosome III (Figure 1C,D). The *MAT* locus contains one of the two alternative alleles, *MATα*—in alpha-type cells or *MATa*—in a-type cells, with diploids being heterozygous *MATa*/*MATα*. The vast majority of laboratory strains are heterothallic strains having a stable haploid and diploid state and mating type during prolonged culture growth. These strains lack the HO endonuclease which initiates mating type switching [171].

For some research purposes, it may be necessary to change the mating type of the strain of interest, for example, when there is a need to use two isogenic strains with opposite mating types, etc. Mating type switching is characteristic of the homothallic yeast strains which naturally express the HO endonuclease. This endonuclease cuts DNA at a specific site in the *MAT* locus [171] and stimulates homologous recombination between the *MAT* locus and one of two silent cassettes *HMRa* or *HMLα* located on the chromosome III [172,173] (Figure 1C), resulting in the replacement of genetic material in the *MAT* locus by an alternative one from the donor sequence, while the cassettes remain intact. If the cell has mating type a, recombination more often occurs between the *MATa* and *HMLα*, and vice versa in alpha mating type cells, where recombination mainly occurs between *MATα* and *HMRa* [173]. The gene conversion between the *MAT* locus and one of the cassettes leads to the mating type switch (Figure 1C). Note that the switch occurs during cell division only in mother cells because the mRNA-encoding Ash1 repressor of HO gene expression is localized in the bud nucleus and inhibits mating type switching in a bud [174].

The most popular and convenient way to switch mating type in yeast is to use plasmids carrying the *HO* gene [175]. These can be integrative or centromeric plasmids with various selective markers (*URA3*, *LEU2*, *kan^r^*, etc.) [176,177]. The expression of the *HO* gene can be constitutive or regulated by galactose or estradiol depending on the promoter used for the *HO* gene expression [171,175,177,178,179,180]. Regardless of the plasmid used, the following steps are taken to switch mating type in a yeast strain [175]. Yeast cells are transformed with a plasmid containing the *HO* gene, and the transformants are selected and then streaked on selective media according to the plasmid’s selective marker. Then, if necessary, the expression of the *HO* gene is induced by transferring the cells to a medium allowing the maintaining of the plasmid and containing an expression inducer (galactose or estradiol). If the *HO* gene on the plasmid is constitutively expressed, then the induction step is skipped. Next, the transformants are passaged on nonselective media (2–3 passages) to lose the plasmid with the *HO* gene and select individual clones. Several clones are selected and tested for their mating type and the HO plasmid presence. Usually, when using a plasmid with inducible *HO* expression, it is possible to select haploid clones that have switched their mating type due to an optimal time of *HO* expression. In the case of constitutive *HO* expression, this becomes more difficult because cells switch their mating type and can quickly become diploid by mating with neighboring cells. In this case, one can select a diploid clone that has lost the *HO*-containing plasmid, sporulate it by incubating cells on media without nitrogen sources, and isolate haploid cells of the desired mating type, using either tetrad dissection or analysis of several random ascospores. Tetrad dissection is preferable in most cases because when a clone is selected from a normal 2a:2α tetrad it ensures that the resulting strain is not spontaneous diploid homozygous for the *MAT* locus. It should be noted though that strains which have been passed through meiosis usually accumulate more mutations compared to mitotically dividing cells, which occurs due to the activation of Spo11, the protein that introduces the meiosis specific DSBs, and subsequent repair of the resultant DSBs [181,182].

When the *HO* gene is expressed from its own promoter, only haploid strains can undergo mating type switching, as the *HO* gene is repressed in heterozygous diploids *MATa*/*MATα* [5]. In rare cases (less than 10^−4^), diploid cells may lose heterozygosity at the *MAT* locus, resulting in homozygous diploids *MATa*/*MATa* and *MATα*/*MATα* [183]. Using an alien promoter, e.g., *GAL1*/*10*, for artificial expression of the *HO* enables mating-type switching in haploid *MATa* and *MATα* strains, as well as in homo- and heterozygous diploids for the mating type locus (*MATa*/*MATα, MATa*/*MATa*, and *MATα*/*MATα*).

Recently, several reports have emerged on the use of the CRISPR/Cas9 system to switch mating type in yeast [184,185]. In these studies, endonuclease Cas9, directed by the gRNA, cleaves the DNA at the boundary between the Y and Z regions of the *MATa* or *MATα* and stimulates HR between one of the two silent cassettes and the *MAT*, resembling the natural HO-dependent mating-type switching mechanism. An efficient switching of mating type was achieved in haploid and diploid strains of *S. cerevisiae* transformed with a plasmid expressing Cas9 and a gRNA. The same approach was used to switch mating type in strains with a synthetic chromosome III lacking the *HMLα* and *HMRa* loci, by transforming cells with a plasmid encoding for gRNA and *CAS9*, along with a DNA fragment carrying the donor *MAT* locus [184]. The use of Cas9 to induce DSBs in the *MAT* locus may be preferable in some cases, for example, when it is necessary to change the mating type in a strain lacking a donor sequence for homologous repair, or in a strain which has stack (*mat-stk*) or inconvertible mutations (*inc-mat*), reducing or preventing mating type switch [186]. One disadvantage of the CRISPR/Cas9 is ineffective or off-target cleavage if a nonoptimal sequence of gRNAs is chosen [184].

## 8. Generation of Karyotype Changes in *S. cerevisiae*

### 8.1. Ploidy Changes

It is known that *S. cerevisiae* can exist in haploid, diploid, triploid, tetraploid, and even higher ploidy states. In fundamental research, diploid and haploid strains are commonly used, while industrial applications may use a wider range of ploidy levels depending on the specific tasks being addressed. Yeast strains of different ploidy alter significantly in a number of morphological and physiological parameters such as cellular size and shape, growth rate, tolerance to environmental stressors, yield of certain metabolites or level of genome instability [185,187,188,189,190,191]. For instance, tetraploids exhibit massive chromosome loss and an altered pattern of recombination relative to diploids [192]. Several approaches for obtaining strains varying in ploidy have been developed [179,185]. In general, these methods feature sequential repeated crossing of strains with opposite mating types, alternating with the stage of the mating-type switching in hybrids; this cycle may be repeated several times [193]. For example, by crossing haploid strains of the a and α mating types, it is possible to obtain a heterozygous *MATa*/*MATα* diploid that is not capable of further crossing. Homozygosity of the *MAT* locus in this diploid can be achieved by *HO* or gRNA/Cas9 expression. Then, resulting diploid strains *MATa*/*MATa* and *MATα*/*MATα* may be crossed and tetraploid strains *MATa*/*MATa*/*MATα*/*MATα* can be selected, and so on. Crossing a diploid strain homozygous for the *MAT* locus with a haploid strain of the opposite mating type will result in a triploid formation. Another known approach is also based on yeast crossing, but its fundamental difference from the works just cited is that primary haploid strain has deletion of the *MAT* locus [194]. The *matΔ* strain is transformed with plasmids carrying either *MATa* or *MATα* and different selective markers. Then strains *matΔ [MATa]* and *matΔ [MATα]* are crossed and a diploid hybrid is selected. After this step both plasmids are lost by passaging yeast on nonselective media, and the obtained strain can be transformed again with a plasmid carrying the *MATa* or the *MATα* information in order to obtain diploid strains *matΔ*/*matΔ [MATa]* and *matΔ*/*matΔ [MATα],* which can be crossed to obtain tetraploids, or those strains may be crossed with haploid strains to obtain triploids. This principle allows for obtaining strains of higher ploidy by repeating the listed steps. It should be noted that strains with a deletion of the *MAT* locus by default have the a mating type, since a-specific genes in yeast are expressed constitutively, and the expression of alpha-specific genes requires an activator encoded by the *MATα1* gene, which is absent in *matΔ* strains (Figure 1D). Therefore, we consider it unnecessary to use a plasmid with *MATa* directly to ensure crossing, although a selective marker of the plasmid facilitates the selection of hybrids.

### 8.2. Generation of Aneuploid Strains

Unlike many living organisms including humans, *S. cerevisiae* is highly tolerant to aneuploidy which can be detected in up to 20% of strain isolates [195,196,197,198]. One can obtain both viable disomics (n + 1) and monosomics (2n − 1) on any of the 16 yeast chromosomes. An RNA-binding translational regulator Ssd1 was recently proposed to be involved in aneuploidy tolerance [199], although its role remains controversial [198].

Aneuploidy may facilitate adaptation to stress or harsh environments, see for review [200,201]. It is especially common in industrial strains where it is thought to be a result of adaptation to industrial conditions, fermentation target products or by-products [200]. Aneuploidy can be a result of a strain’s adaptation to different drugs and confer drug resistance that has perspectives in biotechnological engineering [202,203]. At the same time aneuploidy may lead to a decrease in viability, apparently due to the imbalance of multiple proteins and the increased burden on protein quality control systems [204,205,206]. Fast accumulation of additional unselected aneuploidies after loss of a single chromosome has also been reported in yeast [207]. Yeast is a convenient model for studying the effects of aneuploidy on the state of proteome and genome stability, including the role of aneuploidy in the evolution of cancerous tumors [208,209,210]. Artificially generated diploids and polyploids usually have a higher rate of genomic instability [211,212]. LOH due to complete or partial chromosome loss increases with the ploidy level in yeast [213].

In yeast, extra chromosomes can be obtained as a result of nondisjunction in meiosis or mitosis, or when nuclear breakdown occurs in heterokaryons. A set of aneuploid strains—disomics with an arbitrary karyotype, carrying a haploid set of chromosomes and several additional chromosomes—can be obtained from the original triploid strain, which, in turn, can be obtained by crossing a diploid homozygous for the *MAT* locus and a haploid of the opposite mating type. When a triploid strain sporulates, all segregants are usually aneuploids carrying a haploid set and 5–12 extra chromosomes [214]. To obtain disomic strains with one particular extra chromosome, a chromosome transfer strategy [204] is used, based on the phenomenon of “exceptional” cytoduction [215]. During mating, if one of the mating partners has a defect in karyogamy (e.g., due to mutation in the karyogamy gene *KAR1*), nuclear fusion does not occur, which results in the occurrence of heterocaryons and, further, cytoductants—cells possessing mixed cytoplasm and the nucleus of one of the mating partners. Cytoductants may be isolated using special selection systems [216]. However, occasionally individual chromosomes are transferred from one nucleus to another during this cytoduction process [215]. When the two mating partners carry different selectable markers at the same genomic location, these rare chromosome transfers can be selected for. We can recommend using two strains for mating, one of which carries a wild-type allele of a selective marker and another one carries a knock-out of this gene with a dominant marker such as *kan^r^* defining resistance to G418. Note that some of the strains obtained by the use of a chromosome transfer procedure may carry one or several extra chromosomes in addition to the one selected for, therefore the karyotype of the resulting strain should be tested additionally with methods such as comparative genomic hybridization or by NGS [195,204].

A specific chromosome may be lost upon deletion of its centromere [217]. The same can also be achieved by using chromosomes with the conditional centromere, that is achieved by integration of the *GAL1* promoter in *cis* to centromere sequences, thus allowing targeted loss of the modified chromosome under specific conditions [218]. To select monosomic aneuploids for a particular chromosome, copies of this chromosome in the original diploid must carry different dominant selectable markers at the same genomic location. The selection of the clones that have lost one copy of the chromosome of interest can be simplified if the target chromosome carries a counterselectable marker such as *URA3*. The frequency of chromosome loss can be increased by chemical and physical agents such as benomyl, methyl methanesulfonate, or ionizing radiation [219]. An entire yeast chromosome can be eliminated by introducing a DSB around the centromere via CRISPR–Cas9 [220].

A targeted method for obtaining (n + 1) disomies was also proposed [221] which combines the use of a conditional centromere allowing a transient block of sister chromatid disjunction in mitosis [218], and an elegant approach for disomic selection among daughter cells. The targeted chromosome carries the *URA3* gene disrupted by the insertion of centromeric plasmid with the *HIS3* marker and internal fragment of *URA3*. The excision of the plasmid sequence due to recombination between direct repeats of *URA3* fragment restores the functional *URA3* allele but eliminates *HIS3*. Thus, a targeted chromosome may carry either functional *URA3*, or functional *HIS3*, but not both, which allows selecting Ura+ His+ segregants as disomics [221].

### 8.3. Generation of Genome Structural Rearrangements and Karyotype Evolution in Yeast

As we discussed in Section 6, CRISPR/Cas9 system can be efficiently used for chromosome construction, the generation of different genome rearrangement events and karyotype evolution. Both uniquely-targeted and multiple concomitant reciprocal translocations can be generated with this system in the yeast genome [222]. Reciprocal translocations can be obtained upon the introduction of two double-strand breaks (DSBs) in two different chromosomes with two distinct gRNAs while using specific homologous donor templates targeting both loci and forcing the trans-repair of the obtained fragments [223]. Multiple translocations can be obtained by targeting dispersed repeated sequences with a single gRNA and endogenous uncut copies of the repeat to be used as donor DNA [223].

Budding yeast tolerates various karyotypic alterations well, which was efficiently used to create genomes with a reduced number of chromosomes. Thus, yeast genomes with their chromosome number reduced to 2 [149], or even to a single linear giant chromosome consisting of sixteen fused native linear chromosomes and one centromere [150], have been created. The same team also created a yeast strain which contained a single circular chromosome [151]. See Section 6.5 for more details.

Genome restructuring can be achieved through the use of recombinase-based methods, such as Cre/loxP or Flp/FRT [86], discussed in Section 5.2. The Synthetic Chromosome Rearrangement and Modification by LoxP-mediated Evolution (SCRaMbLE) system provides a novel platform for generating various genomic structural rearrangements [224,225,226,227,228,229,230,231,232,233]. It can be used in synthetic genomes such as that of Sc2.0 [234,235], or genomes with synthetic chromosomes, containing multiple symmetric *loxP* sites [236]. For instance, such *loxP* sites can be introduced 3 bp downstream of stop codons of all nonessential open reading frames (ORFs) and in place of deleted non-intronic features [234]. Upon induction of the Cre recombinase these sites recombine with each other, which ensures stochastic generation of various genome changes including deletions, duplications, inversions and translocations. Substantially, SCRaMbLE represents the inducible evolution system for the generation of large genotypic diversity, which can be followed by screening for advantageous changes. SCRaMbLEing of a synthetic yeast ring chromosome facilitates genome rearrangements and allows the generation of a broad variety of structural variations [237].

Other methods for the generation of complex structural genome rearrangements use temperature-dependent nucleases, such as Taq1, which recognizes the TCGA palindrome and can theoretically cleave genomic DNA once every 256 base pairs [238].

Entering the meiotic program in diploid strains and Return To mitotic Growth (RTG) induced Spo11-dependent DSB formation and their consequent repair associated with intensive LOH [239]. RTG allows recombination in *Saccharomyces* hybrids that are sterile due to common reproductive barriers [240]. This approach has been recently used to stimulate genome shuffling in sterile industrial polyploid strains in order to select for improved biotechnological traits [241].

Natesuntorn et al. developed a PCR-mediated chromosome duplication (PCDup) technique that enables the generation of segmental duplications at any desired chromosomal region [242].

Earlier we have shown that the insertion of short tracts of yeast telomeric repeats to a specific internal chromosomal site can induce translocations, deletions, inversions upon recombination with telomere and even formation of a minichromosome [243,244].

## 9. Conclusions

The use of *S. cerevisiae* as a model organism has made an incredible contribution to fundamental science and has allowed the understanding of the basic molecular processes in eukaryotic cells, such as biosynthesis and energy metabolism, replication, maintenance and expression of genetic material, cell cycle and cell division control, etc. Furthermore, the use of yeast as a model organism has also contributed significantly to applied research, such as the production of biofuels and different pharmaceutical compounds. All of this has become possible largely due to the development of numerous methods for yeast genome modification, which have reached their peak nowadays. Methods available now allow the construction of a yeast strain with any genome modification, either single nucleotide substitution, or chromosome number change, or even multiple genome changes within a few days or weeks. Choosing the most appropriate method is determined by a number of factors, such as the ability to use selective markers, the availability of plasmids and vectors, time and labor costs etc. The type of yeast strain being used can also impact the choice of method. Different strains may have varying levels of genetic stability, growth rates, and susceptibility to certain manipulations. Additionally, some genome-modification methods pose a danger of uncontrolled mutagenesis in the genome. Thus, genome-editing methods using CRISPR/Cas systems allow in a short time for the introduction of multiple changes in the genome; however, this is often accompanied by the occurrence of non-specific genome changes, which can affect the physiology and phenotype of the strain and can be detected only by whole-genome sequencing. For this reason, CRISPR/Cas-based methods can be successfully used, for example when constructing strains for biotechnology requiring multiple genome changes but not sensitive to occasional mutations. At the same time, in fundamental scientific experiments requiring a strict assessment of the effect of a particular mutation or the deletion of a particular gene, the use of classical methods for changing the genome, the action of which is limited only by the modification site, is, as a rule, more relevant. Ultimately, the researcher must carefully weigh all of these factors and choose the method that is most likely to yield reliable and informative results. With the right approach, yeast can continue to be a valuable tool for fundamental and applied research.

## Data Availability

No new data were created or analyzed in this study. Data sharing is not applicable to this article.
